# Identification of prognostic immune-related lncRNAs in pancreatic cancer

**DOI:** 10.3389/fimmu.2022.1005695

**Published:** 2022-11-07

**Authors:** Yan Ma, Xiaomeng He, Yang Di, Shanshan Liu, Qilin Zhan, Zhihui Bai, Tianyi Qiu, Christopher Corpe, Jin Wang

**Affiliations:** ^1^ Shanghai Public Health Clinical Center, Fudan University, Shanghai, China; ^2^ Department of Pancreatic Surgery, Huashan Hospital, Fudan University, Shanghai, China; ^3^ Institute of Clinical Science, Zhongshan Hospital, Shanghai Medical College, Fudan University, Shanghai, China; ^4^ Nutritional Science Department, King’s College London, London, United Kingdom

**Keywords:** pancreatic cancer, immune infiltration, prognostic model, lncRNAs, irlncRNAs

## Abstract

Long noncoding RNAs (lncRNAs) play a critical role in the immune regulation and tumor microenvironment of pancreatic cancer (PaCa). To construct a novel immune-related prognostic risk model for PaCa and evaluate the prognostic prediction of lncRNAs, essential immune-related lncRNAs (IRlncRNAs) were identified by Pearson correlation analysis of differentially expressed immune-related genes (IRGs) and IRlncRNAs in PaCa from The Cancer Genome Atlas (TCGA) and GTEx databases. Least absolute shrinkage and selection operator (LASSO) regression was also applied to construct a prognostic risk model of IRlncRNAs, and gene set enrichment analysis (GSEA) was further applied for functional annotation for these IRlncRNAs. A total of 148 IRlncRNAs were identified in PaCa to construct a prognostic risk model. Among them, lncRNA LINC02325, FNDC1-AS1, and ZEB2-AS1 were significantly upregulated in 69 pairs of PaCa tissues by qRT−PCR. ROC analyses showed that LINC02325 (AUC = 0.80), FNDC1-AS1 (AUC = 0.76), and ZEB2-AS1 (AUC = 0.75) had a good predictive effect on 5-year survival prognosis. We demonstrated that high expression levels of ZEB2-AS1 and LINC02325 were not only positively associated with tumor size and CA199, but elevated levels of ZEB2-AS1 and FNDC1-AS1 were also positively correlated with tumor stage. GSEA further revealed that immune-related pathways were mainly enriched in the high-risk groups. Several immune-related algorithms demonstrated that four IRlncRNAs were related to immune infiltration, immune checkpoints, and immune-related functions. Thus, the prognostic risk model based on IRlncRNAs in Paca indicates that the four IRlncRNA signatures may serve as predictors of survival and potential predictive biomarkers of the pancreatic tumor immune response.

## Introduction

Pancreatic cancer (PaCa) is a highly malignant tumor, with pancreatic ductal adenocarcinoma (PDAC) accounting for 85% of all types of pancreatic cancer (1). The five-year survival rate of patients is less than 5%, and the incidence rate has been steadily increasing in recent years (2). Family history is known to be an independent risk factor for pancreatic cancer. Other risk factors include sex, age, smoking, and diabetes (3). Most patients with pancreatic cancer are asymptomatic until the disease develops to an advanced stage, and when the tumor invades surrounding tissues or has distant metastasis, it is already at an advanced stage (4). Over the past few years, new insights into the tumor microenvironment (TME) have shown that tumor-infiltrating immune cells play an essential role in tumor progression and invasion (5, 6). Cancer-associated fibroblasts (CAFs), as a crucial component of the TME, can interact with immune infiltrating cells to enable cancer cells to acquire immune privilege and evade the surveillance of the immune system (7). The long noncoding RNA (lncRNA) UPK1A-AS1 could be induced by cancer-associated fibroblasts and confer chemoresistance in pancreatic cancer (8). On the other hand, tumor immune escape is an important strategy for tumor progression and leads to the failure of immunotherapy (9). The occurrence of immune escape is mainly attributed to two immune factors: immunosuppressive cells and immunosuppressive molecules (9). Immune suppression can be driven by enhancing the activation of regulatory T cells (Treg), myeloid-derived suppressor cells (MDSCs) and M2 macrophages (10). Programmed death ligand-1/programmed death-1 (PD-L1/PD-1) and CTLA-4, also known as immune checkpoints, are the most common immunosuppressive molecules that can suppress the activation of effector T lymphocytes, ultimately promoting tumor evasion (11). The upregulated lnc01140 in lung cancer could facilitate tumor escape by protecting PD-L1 mRNA from miRNA-mediated inhibition (12). LncRNA KCNQ1OT1 derived from tumor exosomes could function as a miRNA sponge to regulate PD-L1 ubiquitination to promote colorectal cancer immune escape (13). Immune infiltrating cells in the tumor microenvironment are increasingly considered to be associated with the progression, treatment, and prognosis of pancreatic cancer patients. An immune score based on the four-density percentile mean of two markers (CD3^+^ and CD8^+^) and two regions (tumor and invasive marginal regions) has been internationally recognized as a risk assessment tool for colon cancer, illustrating the potential importance of evaluating tumor immune infiltration in guiding clinical decision making (14). However, the traditional immunohistochemical immune scoring methods are still unsatisfactory in PaCa due to the lack of consistent criteria and limited biomarkers for evaluation.

Long noncoding RNAs are longer than 200 nucleotides and do not translate into functional proteins, accounting for more than 80% of the total RNA (15). LncRNAs are uniquely expressed in various normal tissues and cancer cells (16). Accumulated evidence suggests that lncRNAs are widely expressed and participate in gene regulation by modulating translation regulation, histone modification, or posttranscriptional processes (16) and are actively involved in tumor biological processes such as H19, PVT1, NEAT1, and HISLA, which have been found to be associated with tumorigenesis, epithelial-mesenchymal transition, metastasis, chemotherapy resistance, immune evasion, and metabolic reprogramming (17–21). LncRNAs can regulate EZH2 expression by targeting miRNAs (17), participate in regulating therapy response and play a key role in immune regulation in prostate cancer (16). Tumor cell-derived exosomes can serve as lncRNA carrier systems to regulate cancer progression and remodel the tumor microenvironment (18). Recent studies have highlighted the role of long noncoding RNAs in the immune system as critical regulators that can control the differentiation and function of immune cells (19–22), and tumor immune infiltration-related lncRNAs play an important role in the tumor immune microenvironment and tumor immune response (23–25). For example, lncRNA NKILA promoted T-cell sensitivity to activation-induced cell death (AICD) by repressing NF-κB and led to the immune escape of lung cancer cells (26), and SATB2-AS1 could affect intratumoral immune cell abundance and cytokines to inhibit colorectal cancer progression (27). Glycolysis-related lncRNAs, including lncRNA MIR4435-2HG, are associated with high immune infiltration and poor prognoses (28). Immune-related lncRNAs also served as independent risk factors for the overall survival of colorectal cancer patients (25). However, research on lncRNAs associated with infiltrating immune cells in the tumor microenvironment is still limited, especially in pancreatic cancer.

To determine the effect of lncRNAs on infiltrating immune cells in pancreatic cancer, in this study, we aimed to develop a novel lncRNA profile associated with TME immune infiltrating cells to predict the prognosis of patients with pancreatic cancer and evaluate the predictive ability of candidate lncRNAs. We identified a variety of lncRNAs associated with TME immune infiltrating cells as potential biomarkers for the prognosis of pancreatic cancer, which will improve the current diagnosis, treatment, follow-up and prevention strategies for pancreatic cancer.

## Materials and methods

### Patients and tissue specimens

PaCa tissues and adjacent tissues from 69 pairs were obtained from the Department of Pancreatic Surgery, Huashan Hospital Affiliated with Fudan University (Shanghai, China). All procedures were authorized by the Ethics Committee of Shanghai Public Health Clinical Center. Fresh tissue samples were stored in liquid nitrogen after surgical resection and packaging until use.

Moreover, we obtained transcriptome profile data of pancreatic cancer patients from The Cancer Genome Atlas Program (TCGA), including a total of 178 pancreatic cancer patients and 4 normal pancreatic tissues, and excluded patient samples with incomplete clinical information. At the same time, 167 cases of mRNA data of normal pancreatic tissues in the GTEx database were downloaded from the UCSC xena (https://xenabrowser.net/datapages/) website. Immune-related genes (IRGs) were collected from IMMPORT Private Data (https://immport.niaid.nih.gov), and pancreatic cancer immune-related lncRNAs (IRlncRNAs) were downloaded from the IMMLNC website (http://biobigdata.hrbmu.edu.cn/ImmLnc/) (29).

### RNA extraction and qRT−PCR analysis

Total RNA was extracted from tissues using TRIzol reagent (Life Technologies, Austin, Texas, USA) according to the manufacturer’s instructions (Invitrogen). RNA was quantified using a NanoDrop 2000c instrument (ThermoFisher). Then, 1 μg of total RNA was reverse transcribed into cDNA using the PrimeScript RT Reagent Kit (Takara, Dalian, China) following the manufacturer’s protocol. Quantitative real-time PCR (qRT−PCR) analysis was performed by using TB Green Premix Ex Taq (Takara, Dalian, China). The lncRNA expression level for each sample was standardized to 18S expression, and three biological repetitions were carried out. The relative expression level of each lncRNA was quantified using the 2^-ΔΔCt^ method. The primer sequences for qRT−PCR are shown in **Table S1**.

### Identification of differentially expressed genes and immune-related lncRNAs/mRNAs in PaCa

The differentially expressed genes (DEGs) in TCGA-PaCa tissues combined with GTEx normal pancreatic tissues were obtained by using the R software package ggplot2. The criteria for significant differential expression were |log_2_(FC)| (fold change) ≥ 2.0 and *P* adj **≤** 0.05. The common genes that were both differentially expressed and immunologically related in PaCa were identified by Venn diagram analysis of these differentially expressed genes and IRGs. Subsequently, DAVID (https://david.ncifcrf.gov/) bioinformatics resources were used to analyze the GO and KEGG enrichment analysis of these genes. The criterion for statistically significant differential expression was a *P* value < 0.05. To further evaluate immune-related lncRNAs IRlncRNAs in PaCa, all IRlncRNAs were downloaded from the ImmLnc website. Pearson correlation analysis was employed to analyze the correlation between these IRlncRNAs and the differentially expressed immune-related genes (IRGs) in PaCa. The screening threshold |Pearson coefficient| > 0.7 and *P* adj ≤ 0.01 were set for the IRlncRNAs in PaCa.

### Definition and evaluation of a prognostic risk model for IRlncRNAs in PaCa

The R software package glmnet was used to integrate the survival time, survival state and gene expression data, and regression analysis was performed using the lasso method. By setting the λ value to 0.100961694402045, the potential candidate IRlncRNAs were determined. Risk scores were calculated using the same formula as in previous studies (30, 31). According to the median risk score, all patients with PaCa were divided into a high-risk group and a low-risk group. The validity of the prognostic model was assessed by using the Kaplan−Meier survival method, while ROC curve analysis was performed using the R software package pROC to acquire the area under the ROC curve (AUC) to further evaluate the prognostic specificity and sensitivity of the model. In addition, Principal component analysis (PCA) was performed using the R software package stats (version 3.6.0). TSNE analysis was carried out by applying the R software package Rtsne (Version 0.15). The R software package UMAP (version 0.2.7.0) was used for UMAP analysis. At the same time, we analyzed the relationship between different risk scores and patients’ follow-up time, survival state and the expression level changes of each gene and drew the relationship between the expression heatmap of related lncRNAs and prognosis score.

### Gene set enrichment analysis of high-risk and low-risk groups and the nomogram analysis of the overall survival in pancreatic cancer patients

Gene set enrichment analysis (GSEA) was applied to analyze crucial functional pathways for the high-risk and low-risk groups in PaCa patients. Two gene sets, c5.go.bp.v7.4.symbols.gmt and c2.cp.kegg.v7.4.symbols.gmt, which were downloaded from the Molecular Signatures Database (http://www.gsea-msigdb.org/gsea/downloads.jsp), were utilized to evaluate the related functional phenotypes and molecular mechanisms. A false discovery rate (FDR) < 0.05 was regarded as statistically significant.

Using the R software package rms, which integrates the data of survival time, survival state and seven characteristics, and using the Cox method, we established a nomogram to predict the overall survival (OS) of pancreatic cancer patients at one year, three years and five years. The prognostic significance of these characteristics was assessed in 176 samples. Additionally, the calibration curve was plotted to present the prognostic prediction performance of the nomogram.

### Evaluation of the immune landscape of the differentially expressed prognostic IRlncRNAs in PaCa

We analyzed the relationship between the differentially expressed prognostic IRlncRNAs and immunocyte characteristics using a variety of currently accepted methods for the assessment of immunocompetent cells, including XCELL, TIMER, QUANTISEQ, MCPOUNTER, EPIC, CIBERSORT- ABS, and CIBERSORT. The relationship between potential candidate IRlncRNAs and infiltrating immune cells was analyzed by Spearman correlation analysis. The results were filtered with a *P* value < 0.05. We obtained 28 immune infiltration datasets and analyzed the infiltration levels of different immune cells by using the ssGSEA method of the R software Gene Set Variation Analysis (GSVA) package. Based on the analyses of ssGSEA, the samples of pancreatic cancer in TCGA database were classified into high immune cell infiltration, median immune cell infiltration and low immune cell infiltration groups using the “hclust” algorithm.

### Statistical analysis

All experiments in this study were performed independently with at least three biological replicates. All results are presented as the means ± SDs (standard deviation) from triplicates. The statistical analyses were performed in GraphPad Prism 9.0 software (GraphPad Software, San Diego, CA, USA). A paired sample *t* test was used to evaluate the difference between two groups. Fisher’s exact test was performed to assess the significance of the clinicopathologic characteristics between the two groups of categorical samples. *p* < 0.05 was considered to indicate statistical significance (* represents *p* < 0.05, ** represents *p* < 0.01, *** represents *p* < 0.001, and **** means p < 0.0001).

## Results

### Identification of differentially expressed immune-related genes and lncRNAs in PaCa

**Figure 1:**
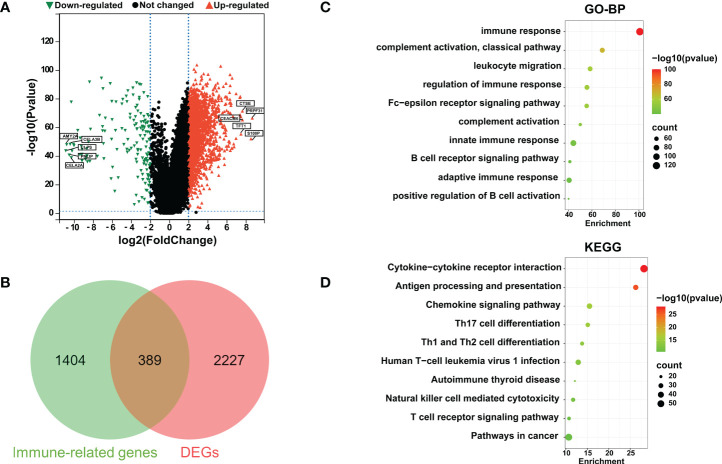
Identification of differentially expressed immune-related genes (IRGs) in PaCa. **(A)** Volcano plots for DEGs in PaCa; red points are up; green points are down. **(B)** A Venn diagram was used to analyze immune-related genes and DEGs in PaCa. **(C)** GO analysis including biological process (BP). **(D)** Kyoto Encyclopedia of Genes and Genomes (KEGG) enrichment analyses for immune-related DEGs. The color represents the *P* value, and the point size denotes the number of genes.

The RNA-seq mRNA expression profiles of 4 normal paracancerous tissues and 178 PaCa tissues were downloaded from the TCGA database, and mRNA data of normal pancreatic tissues from the GTEx database were obtained from the UCSC xena website (https://xenabrowser.net/datapages/), totaling 167 cases. Using |log_2_FC| > 2.0 and *P* adj < 0.05 as the threshold criteria, 2616 DEGs were accumulated, of which 2000 genes were upregulated, and 616 genes were downregulated (**Figure 1A**). Subsequently, a total of 1793 IRGs were collected from the IMMPORT database, Venn diagram analysis was performed on the 1793 IRGs and 2616 DEGs, and a total of 389 genes that were differentially expressed in PaCa and related to immunity were identified, indicating that these genes might play a vital role in the immune regulation of PaCa (**Figure 1B**). Then, we performed GO and KEGG enrichment analyses of these differentially expressed IRGs and found that these IRGs were highly enriched in several immune-related biological processes, including immune response, complement activation, classical pathway, regulation of immune response, innate immune response, B-cell receptor signaling, adaptive immune response and positive regulation of B-cell activation (**Figure 1C**). Similarly, KEGG enrichment analysis revealed that these IRGs were mainly enriched in immune-related signaling pathways, such as cytokine−cytokine receptor interaction, antigen processing and presentation, chemokine signaling pathways, and T-cell receptor signaling pathways (**Figure 1D**). To further evaluate immune-related lncRNAs in PaCa, Pearson correlation analysis was performed on 4126 IRlncRNAs derived from ImmLnc and 389 IRGs derived from Venn diagram analysis. Under the conditions that |Pearson coefficient| > 0.7 and *P* adj ≤ 0.01, 148 IRlncRNAs closely related to PaCa were obtained, and 23 significant IRlncRNAs and correlated genes were obtained by |log_2_FC| ≥ 2.0 and *P* < 0.001, as shown in **Table 1**. A flowchart of our analysis process is shown in **Figure S1**.

**Table 1:** Partial differentially expressed immune-related genes (IRGs) and immune-related lncRNAs (IRlncRNAs) in PaCa based on correlation analysis.

LncRNA symbol	LncRNA Ensembl	lncRNA		Gene name	Accession number	Gene		Correlation	
						Log_2_FC	*P* value			Log_2_FC	*P* value	R	*P* value
LINC01133	ENSG00000224259	4.56	7.55E-56	CTSE	NM_001317331.2	7.66	6.63E-66	0.78	6.28E-35
RP11-350J20.12	ENSG00000273132	4.262	1.03E-58	S100A6	NM_005514.8	6.572	4.1E-95	0.74	3.24E-28
MIR4435-2HG	ENSG00000172965	3.64	5.07E-71	PLAU	NM_001145031.3	4.88	2.84E-71	0.73	2.07E-28
ZEB2-AS1	ENSG00000238057	3.27	4.90E-12	GMFG	NM_001301008.2	8.66	9.67E-58	0.70	8.34E-25
HCP5	ENSG00000206337	3.08	3.21E-59	HLA-B	NM_005514.8	3.83	1.74E-63	0.78	3.25E-27
PCED1B-AS1	ENSG00000247774	2.75	2.05E-46	C3AR1	NM_001326475.2	2.25	1.51E-37	0.71	9.44E-26
PSMB8-AS1	ENSG00000204261	2.68	1.40E-57	HLA-E	NM_005516.6	2.75	2.63E-57	0.73	1.92E-28
LINC01614	ENSG00000230838	2.62	1.20E-47	EDNRA	NM_001166055.2	3.21	9.29E-71	0.76	8.50E-31
LINC01272	ENSG00000224397	2.60	2.06E-49	FCER1G	NM_004106.2	3.37	2.06E-47	0.71	4.56E-28
ABHD11-AS1	ENSG00000225969	2.44	2.17E-33	MAPK3	NM_001040056.3	2.74	1.07E-78	0.71	9.90E-26
BX470102.3	ENSG00000238279	2.38	7.82E-42	S100A6	NM_014624.4	6.57	4.10E-95	0.79	3.83E-36
FNDC1-AS1	ENSG00000233682	2.28	2.45E-39	NOX4	NM_001143836.3	7.63	1.56E-61	0.74	4.30E-29
RP11-304L19.3	ENSG00000261123	2.11	1.87E-38	MAPK3	NM_001040056.3	2.74	1.07E-78	0.70	4.30E-29
TEX26-AS1	ENSG00000224743	2.07	1.38E-07	THBS1	NM_003246.4	6.77	8.43E-28	0.70	9.13E-25
LINC02325	ENSG00000246084	2.05	4.38E-11	CD3E	NM_000733.4	7.47	6.46E-45	0.73	4.50E-28
ROCR	ENSG00000228639	-2.16	7.49E-43	REG1A	NM_002909.5	-5.95	9.79E-33	0.77	4.20E-32
LYPLAL1-AS1	ENSG00000228536	-2.41	7.51E-39	REG1A	NM_002909.5	-5.95	9.79E-33	0.72	5.62E-27
MIR217HG	ENSG00000226702	-2.71	1.06E-64	GPHA2	NM_130769.4	-4.20	1.24E-44	0.74	4.20E-32
ERVE-1	ENSG00000267259	-3.16	7.80E-91	PDIA2	NM_006849.4	-6.76	5.39E-58	0.71	1.24E-25
LINC00671	ENSG00000213373	-3.37	1.10E-59	PDIA2	NM_006849.4	-6.76	5.39E-58	0.79	1.91E-36
LINC01829	ENSG00000236780	-3.63	4.68E-62	PDIA2	NM_006849.4	-6.76	5.39E-58	0.73	1.57E-28
RP11-320N7.2	ENSG00000256969	-3.67	1.15E-47	GPHA2	NM_130769.4	-4.20	1.24E-44	0.77	3.47E-33
RP11-680B3.2	ENSG00000240521	-4.97	5.26E-77	GPHA2	NM_130769.4	-4.20	1.24E-44	0.77	8.69E-33

### Establishment of a specific prognostic risk model based on the differentially expressed IRlncRNAs in pancreatic cancer

**Figure 2:**
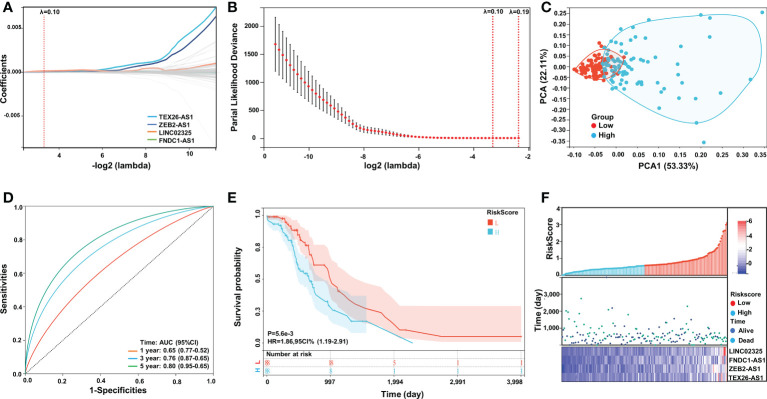
Construction and validation of the IRlncRNA prognostic risk model by LASSO regression analysis. **(A, B)** LASSO regression was performed with the minimum criteria. LASSO coefficient profiles of four prognostic IRlncRNAs (LINC02325, FNDC1-AS1, ZEB2-AS1, TEX26-AS1). **(C)** PCA plot. **(D)** ROC curve of the four-IRlncRNA signature for 1-, 3-, and 5-year survival in the TCGA cohorts. **(E)** Kaplan−Meier curves for the OS of PaCa patients in the high- and low-risk groups. **(F)** Risk score distribution, survival status and expression of four IRlncRNAs in PaCa patients.

To construct an IRlncRNA-based prognostic model for PaCa patients, 148 IRlncRNAs were incorporated into the least absolute shrinkage and selection operator (LASSO) regression analysis. When λ = 0.10, a total of 4 prognosis-related IRlncRNAs were obtained, including LINC02325, FNDC1-AS1, ZEB2-AS1 and TEX26-AS1 (**Figures 2A, B**). According to the median risk score (the risk score is 0.57314892), the patients with PaCa were divided into a high-risk group (n = 88) and a low-risk group (n = 88) (**Table S2**). Principal component analysis showed that the distribution differences between the high-risk and low-risk subgroups were significant (**Figure 2C**). Similarly, t-SNE and UMAP analysis also proved the statistical rationality of the prognostic model (**Figures S2A, B**). The ROC curve analysis revealed that these four IRlncRNAs had superiority in predicting the prognosis of patients with 1-year, 3-year, and 5-year disease, with AUC values of 0.65, 0.76, and 0.80, respectively (**Figure 2D**). Kaplan−Meier survival analysis showed that the OS of PaCa patients in the high-risk group was significantly lower than that in the low-risk group (*P* < 0.001, **Figure 2E**). Moreover, most of the deaths were mainly distributed in the high-risk group. The heatmap of the expression profiles of differentially expressed IRlncRNAs in PaCa indicated that LINC02325, FNDC1-AS1, ZEB2-AS1, and TEX26-AS1 were all highly expressed in the high-risk group. (**Figure 2F**).

**Table 2:** Correlations between four IRlncRNA expression levels and clinicopathological characteristics in 69 PaCa patients.

Clinicopathologic feature	ZEB2-AS1		*P* value	LINC02325		*P* value	FNDC1-AS1		*P* value	TEX26-AS1		*P* value
		High expression		Low expression		High expression	Low expression		High expression	Low expression		High expression	Low expression	
**All cases**	46	23		50	19		37	32		31	38	
**Age**												
≤ 60	15	10	0.432	18	12	0.058	14	11	0.806	9	16	0.319
> 60	31	13	32	7		23	21		22	22	
**Gender**												
Male	30	14	0.793	33	11	0.100	17	27	0.001**	18	26	0.453
Female	16	9	17	8		20	5		13	12	
**Diameter of tumor (cm)**												
≤ 4	14	9	0.022*	11	10	0.021*	20	20	0.800	18	22	>0.999
> 4	31	14	38	9		17	11		13	15	
**Tumor stage**												
I / II	17	15	0.011*	26	13	0.061	7	14	0.017*	21	18	0.287
III/IV	24	4	19	2		26	13		8	13	
**Ki67 positive rate (%)**												
≤ 10	12	7	0.360	16	3	0.737	11	8	0.593	10	9	0.582
> 10	32	10	32	10		21	21		18	24	
**CA199 (U/ml)**												
≤ 37	4	16	<0.0001****	4	6	0.027*	7	3	0.493	3	7	0.314
> 37	40	6	43	13		30	26		28	28	
**CA125 (U/ml)**												
≤ 35	35	15	0.712	36	15	0.712	28	23	>0.999	24	27	>0.999
> 35	9	2	9	2		6	5		5	7	

Bold values represent statistical significance.

* represents p < 0.05, ** represents p < 0.01, *** represents p < 0.001, and **** means p < 0.0001.

### Correlation analysis of differentially expressed IRlncRNAs and clinical pathological characteristics of PaCa

**Figure 3:**
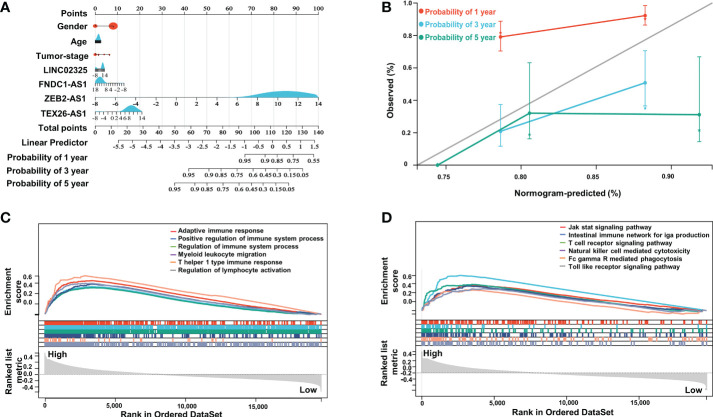
Nomogram analysis of four IRlncRNAs and GSEA between the two subgroups. **(A)** Nomogram of the clinical prognostic model based on sex, age, stage, and the expression of four IRlncRNAs. **(B)** Calibration curves for the 1-, 3- and 5-year survival plots comparing the actual and predicted values. **(C)** GSEA indicated significant enrichment of immune-related phenotypes in high-risk PaCa patients based on c5.go.bp.v7.4.symbols.gmt; **(D)** was based on c2.cp.kegg.v7.4.symbols.gmt.

To further evaluate the prognostic effects of these IRlncRNAs, we divided the patients into different subgroups based on sex, age and tumor stage and compared the risk score levels among different groups. Our results showed that the risk scores of patients in clinical stage III were higher than those in clinical stage I (**Figure S3C**), although there was no significant difference in the risk scores of patients of different sexes and ages (age was divided by 60 years) (**Figures S3A, B**), which demonstrated that differentially expressed IRlncRNAs in PaCa might have certain predictive value for clinical staging. Furthermore, to assess the survival probability of patients, we constructed a nomogram survival prediction model containing four IRlncRNA markers and clinicopathological features (including sex, age, and tumor stage) (**Figure 3A**). The calibration curves for the 1-, 3- and 5-year overall survival plots performed well, indicating that the nomogram model had good accuracy (**Figure 3B**). This nomogram predicts the probability of 3- to 5-year OS for PaCa patients, providing a quantitative method to predict OS for patients and helping clinicians make medical decisions and follow-up plans, which collectively demonstrates that the four IRlncRNA signature may act as an independent prognostic factor for patients with PaCa.

### Gene set enrichment analysis between high- and low-risk groups in PaCa

To investigate remarkable changes in functional phenotypes between the high-risk and low-risk groups, intergroup GSEA was next performed to evaluate functional pathways and molecular mechanisms based on the GO-BP and KEGG molecular signal datasets. Our results showed that immune-related responses were mainly enriched in high-risk groups compared to low-risk groups in the GO-BP annotation dataset, including adaptive immune response, positive regulation of immune system process, T-helper 1 type immune response and regulation of lymphocyte activation (**Figure 3C**). Specifically, immune-related pathways were highly enriched in the high-risk groups, such as the intestinal immune network for IgG production, T-cell receptor signaling pathway, natural killer cell-mediated cytotoxicity, and Toll-like receptor signaling pathway in KEGG molecular signal datasets (**Figure 3D**).

### Validation of differentially expressed IRlncRNAs (LINC02325, FNDC1-AS1, and ZEB2-AS1) as predictive prognostic factors in patients with PaCa

**Figure 4:**
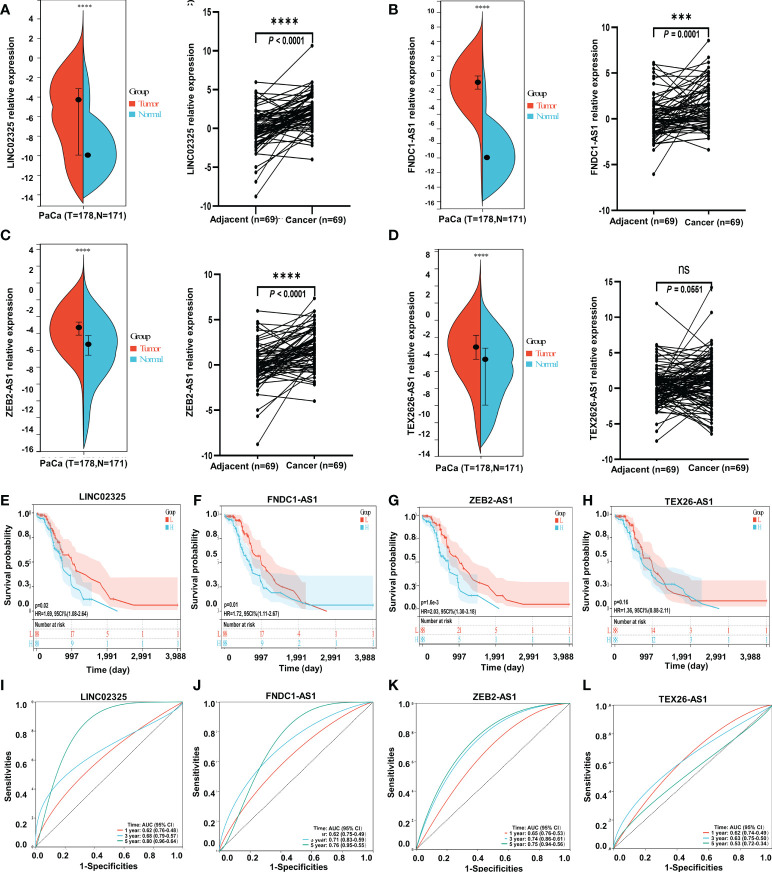
The expression levels of four IRlncRNAs and confirmation of the four IRlncRNA prognostic signatures’ prediction ability. **(A)** LINC02325. **(B)** FNDC1-AS1. **(C)** ZEB2-AS1. **(D)** TEX26-AS1. *** means *P* < 0.001, “ns” means no significance. “****” means *p* < 0.0001. **(E–H)** Kaplan−Meier curve analysis of the correlation between the expression of the four IRlncRNAs and the prognosis of PaCa. **(I–L)** ROC curves for the prognostic prediction of four IRlncRNAs at 1-, 3-, and 5-year survival times.

We further analyzed the expression levels of four IRlncRNAs in normal pancreatic samples and tumor samples based on GTEx and TCGA datasets. Our results suggested that LINC02325, FNDC1-AS1, ZEB2-AS1 and TEX26-AS1 were upregulated in tumor tissues (**Figures 4A–D**). To clarify whether these four IRlncRNAs were specifically expressed in PaCa, we analyzed the four IRlncRNA expression levels in the ten most frequent cancers, including breast cancer, lung cancer, colorectal cancer, prostate cancer, stomach cancer, liver cancer, cervical cancer, esophageal cancer, thyroid cancer, and bladder cancer (https://gco.iarc.fr/today/home). We found that both LINC02325 and FNDC1-AS1 were upregulated in breast cancer and stomach cancer (**Figures S4C, D**), LINC02325 was upregulated in cervical cancer, colorectal cancer and esophageal cancer (**Figure S4C**) and FNDC1-AS1 was upregulated in bladder cancer and lung cancer (**Figure S4D**). However, FNDCA-AS1, ZEB2-AS1 and TEX26-AS1 were downregulated in cervical cancer and colorectal cancer (**Figure S4D**). Moreover, ZEB2-AS1 and TEX26-AS1 were downregulated in all ten cancers (**Figures S4A, B**). To further validate the expression level of these four IRlncRNAs in PaCa, qRT−PCR was conducted to determine the expression level of lncRNAs in 69 pairs of pancreatic cancer and adjacent normal tissues. Our results demonstrated that LINC02325, FNDC1-AS1 and ZEB2-AS1 were more highly significantly expressed in pancreatic cancer tissues than in adjacent normal tissues (**Figures 4A–C**), although there was no significant difference in TEX26-AS1 expression levels in pancreatic tumor tissue (**Figure 4D**). In addition, we analyzed the correlation between the expression of four IRlncRNAs and the clinicopathological characteristics of 69 PaCa patients (**Table 2**). We found that high expression of ZEB2-AS1 was positively associated with tumor size (*P* = 0.022), tumor stage (*P* = 0.011) and CA199 (*P* < 0.0001). The high expression of LINC02325 was positively associated with tumor size (*P* = 0.021) and CA199 (*P* = 0.027). Statistical analyses showed that the high expression of FNDC1-AS1 was positively correlated with tumor stage (*P* = 0.017). Furthermore, the Kaplan−Meier survival analysis showed that the higher the expression of LINC02325, FNDC1-AS1 and ZEB2-AS1 was, the worse the prognosis of patients (**Figures 4E–G**), although TEX26-AS1 had no significant effect on the prognosis of PaCa patients (**Figure 4H**). Moreover, ROC analysis of 1-, 3- and 5-year follow-up times and the expression of four IRlncRNAs showed that LINC02325 (AUC = 0.80; **Figure 4I**), FNDC1-AS1 (AUC = 0.76) (**Figure 4J**), and ZEB2-AS1 (AUC = 0.75) (**Figure 4K**) had a good predictive effect on 5-year survival prognosis. FNDC1-AS1 (AUC = 0.71) (**Figure 4J**) and ZEB2-AS1 (AUC = 0.74) (**Figure 4K**) also had a high-performance predictive effect on 3-year survival, although TEX26-AS1 had poor predictive performance for survival outcomes at 1, 3 and 5 years (**Figure 4L**). We also analyzed other biomarkers related to PaCa survival performance and found that LINC02325 (AUC = 0.80), FNDC1-AS1 (AUC = 0.76), ZEB2-AS1 (AUC = 0.75) had a predictive effect on the 5-year survival prognosis of pancreatic cancer compared with the AUC of these candidate biomarkers, such as LINC01638 (AUC = 0.58), ABHD-AS1 (AUC = 0.61), CCDC26 (AUC = 0.57), HULC (AUC = 0.57), and other miRNAs (miR-25-3p, miR-210-3p, miR-221-3p, and miR-19a-3p) (AUC = 0.53 - 0.62) (**Table 3**).

**Table 3:** The prognostic value of other candidate markers and four IRlncRNAs for pancreatic cancer survival analysis.

Candidate marker	Expression	1-year AUC (95%CI)	3-year AUC (95%CI)	5-year AUC (95%CI)
miR-25-3p	Up	0.59 (0.72-0.46)	0.58 (0.72-0.43)	0.60 (0.81-0.40)
miR-210-3p	Up	0.57 (0.70-0.45)	0.62 (0.84-0.40)	0.62 (0.84-0.40)
miR-221-3p	Up	0.60 (0.72-0.47)	0.53 (0.69-0.38)	0.53 (0.77-0.29)
miR-19a-3p	Up	0.62 (0.76-0.48)	0.47 (0.61-0.43)	0.60 (0.71-0.48)
LINC01638	Up	0.58 (0.71-0.45)	0.67 (0.80-0.43)	0.58 (0.97-0.73)
SNHG15	Up	0.54 (0.67-0.41)	0.60 (0.73-0.46)	0.79 (0.91-0.67)
ABHD-AS1	Up	0.58 (0.70-0.45)	0.64 (0.79-0.49)	0.61 (0.89-0.34)
CCDC26	Up	0.66 (0.78-0.54)	0.54 (0.67-0.42)	0.57 (0.76-0.39)
HULC	Down	0.50 (0.64-0.37)	0.59 (0.73-0.45)	0.57 (0.73-0.41)
ZEB2-AS1	Up	0.65 (0.76-0.53)	0.74 (0.86-0.61)	0.75 (0.94-0.56)
LINC02325	Up	0.62 (0.76-0.48)	0.68 (0.79-0.57)	0.80 (0.96-0.64)
FNDC1-AS1	Up	0.62 (0.75-0.49)	0.71 (0.83-0.59)	0.76 (0.95-0.55)
TEX26-AS1	Up	0.62 (0.74-0.49)	0.63 (0.75-0.50)	0.53 (0.72-0.34)

### Correlation analysis of four prognostic IRlncRNAs with the immune landscape in PaCa

**Figure 5:**
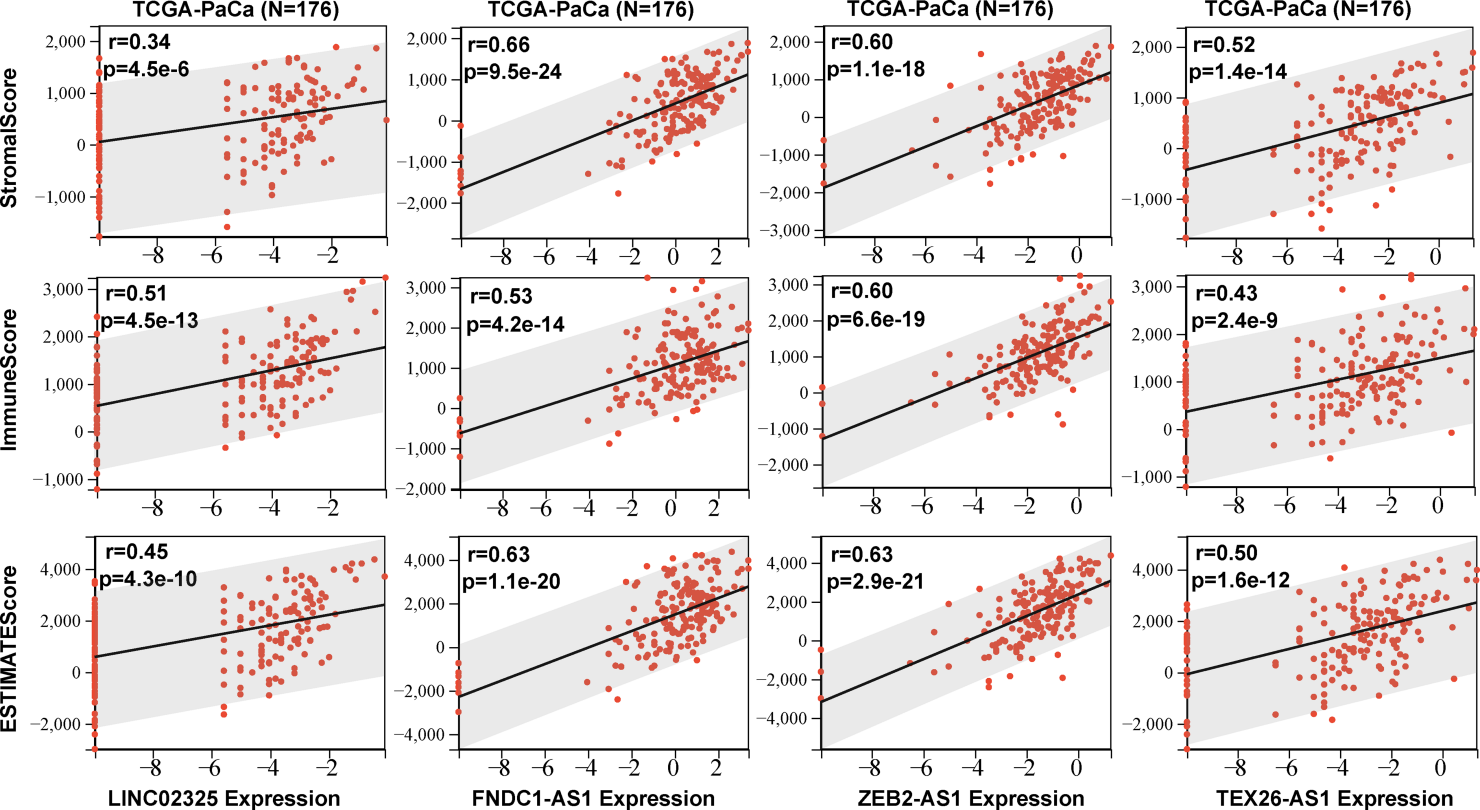
The stromal score, immune score and ESTIMATE score were obtained according to four IRlncRNAs (LINC02325. FNDC1-AS1. ZEB2-AS1. and TEX26-AS1) expression levels.

Four IRlncRNAs identified by correlation analysis and prognostic modeling implied that they might play an important role in the immune signaling response. Four IRlncRNA expression levels were found to be positively correlated with coexpressed IRGs (**Figures S5A–D**). To estimate the content of immune cell infiltration and the tumor microenvironment affected by these prognostic IRlncRNAs, we uploaded lncRNA transcriptome data to eight commonly used immune cell infiltration algorithm resources, including ESTIMATE, TIMER, CIBERSORT, QUANTISEQ, MCPcounter, IPS, Xcell, and EPIC. The ESTIMATE algorithm showed that the expression of these IRlncRNAs was positively correlated with stromal score, immune score, and ESTIMATE score, including LINC02325 (stromal score: r = 0.34, *P* < 0.001; immune score: r = 0.51, *P <* 0.001; ESTIMATE score: r = 0.45, *P* < 0.001), FNDC1-AS1 (stromal score: r = 0.66, *P* < 0.001; immune score: r = 0.53, *P <* 0.001; ESTIMATE score: r = 0.63, *P* < 0.001), ZEB2-AS1 (stromal score: r = 0.60, *P* < 0.001; immune score: r = 0.60, *P <* 0.001; ESTIMATE score: r = 0.63, *P* < 0.001), and TEX26-AS1 (stromal score: r = 0.52, *P* < 0.001; immune score: r = 0.43, *P* < 0.001; ESTIMATE score: r = 0.50, *P* < 0.001) (**Figure 5**), which showed that the tumor purity of the high-expression IRlncRNA group was lower than that of the low-expression IRlncRNA group. Moreover, the TIMER and EPIC algorithms revealed that four IRlncRNAs were markedly associated with the infiltration of various immune cells in PaCa, including B cells, CD4^+^ T cells, CD8^+^ T cells, neutrophils, macrophages and DCs (*P* < 0.05) (**Figures S6A, B**). Finally, the CIBERSORT algorithm was used to analyze the correlation between IRlncRNA expression levels and 22 different immune cell types, including primitive B cells, memory B cells, and plasma cells, in PaCa patients (**Figure S6C**) and other immune cell infiltration algorithms, including MCPcounter, Xcell, QUANTISEQ, IPS, and immune checkpoints (**Figures S7A, B**; **S8A–C**), which suggested that four prognostic IRlncRNAs might be involved in immune cell infiltration and the immune microenvironment in pancreatic cancer. Moreover, 182 pancreatic cancer samples were assigned into three clusters based on the enrichment levels of 28 immune cell infiltrations by performing ssGSEA, including the high immune cell infiltration group (n = 6), median immune cell infiltration group (n = 28) and low immune cell infiltration (n = 148). Our results demonstrated that pancreatic cancer patients with high immune cell infiltration had higher expression levels of four IRlncRNAs (**Figure S9**).

## Discussion

Pancreatic cancer is a highly heterogeneous malignancy characterized by an immune suppressive stromal reaction that creates a barrier to therapy (22, 32). Immune strategies targeting the tumor microenvironment have become a promising approach for the treatment of malignant tumors (33). However, the role of single or combined immune checkpoint inhibitors in pancreatic cancer is still a major clinical challenge, partly due to less infiltration of immune cells, poor immunogenic immune microenvironment and large numbers of mesenchymal fibroblasts blocking drug delivery (34).

To identify reliable and effective biomarkers for PaCa prognosis, we first constructed a prognostic risk model to screen previously unidentified immune-related lncRNAs, including LINC02325, FNDC1-AS1, ZEB2-AS1 and TEX26-AS1 GO and KEGG functional enrichment analyses revealed that these four IRlncRNAs and coexpressed mRNAs were significantly enriched in various immune-related biological processes. Based on the assumption that IRlncRNAs may be essential for basic immune cell differentiation and functions, several algorithms were applied to evaluate the correlation between IRlncRNAs and infiltrating immune cells. Among these four IRlncRNAs, three lncRNAs have not been reported, namely, LINC02325, FNDC1-AS1 and TEX26-AS1, so the specific mechanism of these lncRNAs needs to be further studied. Notably, however, ZEB2-AS1 has been identified in a variety of cancers and may be a potential biomarker (35–37). For instance, ZEB2-AS1 could enhance epithelial-mesenchymal transition through the miR-1205/CRKL pathway in colorectal cancer (38) and may be involved in the occurrence and development of colon cancer by regulating the glycolysis process through the miR-188/TAB3 pathway (39). In addition, in a comprehensive analysis of the microenvironment of lung adenocarcinoma, the study revealed an immune-related lncRNA, ZEB2-AS1, associated with the prognosis of the patient (40). In human pancreatic cancer, lncRNA ZEB2-AS1 could affect cancer cell growth and invasion by regulating the miR-204/HMGB1 axis (41). These studies support our findings that ZEB2-AS1 may be a good reflection of immune cell infiltrates and a novel biomarker.

Although the immunosuppressive state as a distinct biologic feature of pancreatic cancer was observed in many patients (42) and the circulating levels of IL-6, IL-18 and TGF-beta2 in the sera have also been reported to reflect the survival probabilities of patients with PaCa (43), our present data outlined a potentially added clinical value, as LINC02325, FNDC1-AS1, and ZEB2-AS1 may be prognostic factors in patients with pancreatic cancer. Moreover, high expression levels of LINC02325, ZEB2-AS1 and FNDC1-AS1 were also positively associated with CA199, tumor size, or tumor stage. Therefore, we established a novel nomogram survival probability prediction model based on the clinicopathological characteristics of pancreatic cancer, which can provide support for clinicians to design personalized treatment. The results identified that high IRlncRNA expression indicates poor prognosis in pancreatic cancer, which is consistent with previous predictions for high-risk groups. To further validate the expression levels of four prognostic IRlncRNAs in PaCa, qRT−PCR was performed to detect the expression levels of these four lncRNAs in 69 pairs of pancreatic cancer tissues and adjacent normal tissues. The expression trend was basically consistent with the prediction of the previous bioinformatic analysis. However, the TEX26-AS1 expression level showed no statistically significant difference between cancer tissues and adjacent tissues. This may be due to a small sample size from the TCGA public database and the lack of a validation cohort and prospective, multicenter, authentic data for further validation. To assess the intratumor heterogeneity in PaCa, we calculated the microsatellite instability (MSI) (44) and mutant-allele tumor heterogeneity (MATH) scores (45) from the available data derived from the TCGA Pan-Cancer database (https://tcga.xenahubs.net) and investigated their correlations with four IRlncRNA expression levels using Pearson correlation (**Figures S10**, **S11**). We found that in pancreatic cancer, ZEB2-AS1 (*P* = 0.035, r = -0.160) expression levels were negatively associated with MSI scores, and ZEB2-AS1 (*P* = 0.013, r = -0.19) and FNDC1-AS1 (*P* = 0.002, r = -0.24) were also negatively correlated with MATH scores in PaCa, which implied that ZEB2-AS1 might have a role in predicting pancreatic cancer heterogeneity.

In summary, our results suggest that several immune-related lncRNAs (LINC02325, FNDC1-AS1, ZEB2-AS1 and TEX26-AS1) may serve as independent prognostic biomarkers for pancreatic cancer, which provides a theoretical basis for determining the therapeutic target of pancreatic cancer and a promising strategy for guiding individualized treatment and improving prognosis prediction. Given the potential role of IRlncRNAs in the process of immune infiltration, the mechanism by which each IRlncRNA regulates the immune response in PaCa needs to be further elucidated. Further understanding and identification of immune infiltration-related lncRNAs may provide a valuable reference for improving tumor immunotherapy and ameliorating PaCa patient prognosis.

## Conclusions

We established a prognostic risk model based on IRlncRNAs and screened four IRlncRNAs that may serve as predictors of survival and potential predictive biomarkers of the tumor immune response in PaCa.

## Data availability statement

The original contributions presented in the study are included in the article/**Supplementary Material**. Further inquiries can be directed to the corresponding author.

## Ethics statement

The studies involving human participants were reviewed and approved by Ethics Committee of Shanghai Public Health Clinical Center. The patients/participants provided their written informed consent to participate in this study.

## Author contributions

All authors read and approved the final manuscript. JW and YM designed the study. YM was mainly responsible for collecting data and performing data analysis. YM and XH carried out the experiments. YM and JW drafted the manuscript. CC and JW revised the manuscript. Final approval of the version to be submitted: JW, YM, XH, YD, SL, QZ, ZB, TQ and CC. All authors contributed to the article and approved the submitted version.

## Funding

This research was supported by a grant from the National Natural Science Foundation of China (81672383), a grant from the Science and Technology Commission of Shanghai (20Y11900700) and a grant from the Special Research Fund of Youan Medical Alliance for the Liver and Infectious Diseases (LM202020).

## Acknowledgments

We thank all authors for their helpful discussion of the manuscript.

## Conflict of interest

The authors declare that the research was conducted in the absence of any commercial or financial relationships that could be construed as a potential conflict of interest.

## Publisher’s note

All claims expressed in this article are solely those of the authors and do not necessarily represent those of their affiliated organizations, or those of the publisher, the editors and the reviewers. Any product that may be evaluated in this article, or claim that may be made by its manufacturer, is not guaranteed or endorsed by the publisher.

## Abbreviations

lncRNA, long noncoding RNA; PaCa, pancreatic cancer; PDAC, pancreatic ductal adenocarcinoma; IRGs, immune-related genes; IRlncRNAs, immune-related lncRNAs; TME, tumor microenvironment; OS, overall survival; FC, fold change; DEGs, differentially expressed genes; GSEA, gene set enrichment analysis; PCA, principal component analysis.
