# Supplementary material for: Identification of prognostic immune-related lncRNAs in pancreatic cancer
Source: Front Immunol. 2022 Nov 7;13:1005695. doi: 10.3389/fimmu.2022.1005695 (PMC9676238; doi:10.3389/fimmu.2022.1005695)
Supplement: Supplementary file 1 [file DataSheet_1.docx]

***Supplementary Materials***

**Identification of Prognostic Immune-related LncRNAs in Pancreatic Cancer**

Yan Ma^1^, Xiaomeng He^1^, Yang Di^2^, Shanshan Liu^1^, Qilin Zhan^1^, Zhihui Bai^1^, Tianyi Qiu^3^, Christopher Corpe^4^, Jin Wang^1, *^

^1^Shanghai Public Health Clinical Center, Fudan University, 2901 Caolang Road, Jinshan District, Shanghai 201508, P.R. China; ^2^Department of Pancreatic Surgery, Huashan Hospital, Fudan University, Shanghai, China; ^3^Institute of Clinical Science, Zhongshan Hospital, Shanghai Medical College, Fudan University, Shanghai 200032, China; ^4^King's College London, London, Nutritional Science Department, 150 Stamford Street, Waterloo, London, SE19NH, United Kingdom.

**Running Title**: Immune-related lncRNAs in PaCa.

**Keywords:** pancreatic cancer, immune infiltration, prognostic model, lncRNAs, IRlncRNAs

***Correspondence should be addressed to:**

Jin Wang, Ph.D.

Shanghai Public Health Clinical Center,

Fudan University,

2901 Caolang Road, Jinshan District,

Shanghai 201508, China;

Ph: 86-21-57036495;

Fax: 86-21-57247094

Email: [wjincityu@yahoo.com](mailto:wjincityu@yahoo.com)

1. **Supplementary Tables**

| **Name of lncRNAs** | **Sequences of Primers (5’-3’)** |
| --- | --- |
| LINC02325 | Forward: CAGCAAGTAAGAAGGAGCTTAGAAA  Reverse: AGTAGTCACAGAACCCATAGGAGG |
| FNDC1-AS1 | Forward: TAACCTGGCTGAACTCACCG  Reverse: TGGCTACTGCACTGGTGTTC |
| ZEB2-AS1 | Forward: GGAGGCAGGACCGTTATTCC  Reverse: AAGATAGGTGGCGCGTGTTT |
| TEX26-AS1 | Forward: CAAGCTGTGAAATGGTGCGG  Reverse: GCAGACTTCTCTCCCTCACG |
| 18S | Forward: GTAACCCGTTGAACCCCATT  Reverse: CCATCCAATCGGTAGTAGCG |

- 1. **Supplementary Table S1.** Sequences of primers of four IRlncRNAs in PaCa for real-time qPCR.
  2. **Supplementary Table S2.** Analysis of **t**he risk score of the patients with PaCa was divided into a low-risk group (blank) and a high-risk group (yellow).

| **Tag** | **Time** | **Status** | **Risk Score** | **Tag** | **Time** | **Status** | **Risk Score** |
| --- | --- | --- | --- | --- | --- | --- | --- |
| TCGA-FB-AAPP-01 | 485 | 0 | 0.012262917 | TCGA-S4-A8RM-01 | 737 | 1 | 0.391308752 |
| TCGA-HZ-7289-01 | 901 | 0 | 0.015204171 | TCGA-H6-8124-01 | 392 | 1 | 0.391420777 |
| TCGA-3A-A9IS-01 | 998 | 1 | 0.026703031 | TCGA-LB-A8F3-01 | 379 | 1 | 0.392798114 |
| TCGA-F2-6880-01 | 289 | 1 | 0.063213107 | TCGA-HV-A5A6-01 | 3989 | 0 | 0.393389834 |
| TCGA-YY-A8LH-01 | 2016 | 1 | 0.101606885 | TCGA-HZ-7926-01 | 526 | 0 | 0.396003581 |
| TCGA-HV-AA8X-01 | 532 | 0 | 0.104275595 | TCGA-3A-A9IN-01 | 2084 | 1 | 0.396018742 |
| TCGA-3A-A9IO-01 | 1942 | 1 | 0.107696646 | TCGA-HZ-A9TJ-01 | 603 | 1 | 0.397485837 |
| TCGA-3A-A9IR-01 | 1542 | 1 | 0.128970626 | TCGA-2L-AAQI-01 | 103 | 0 | 0.398538617 |
| TCGA-US-A77G-01 | 12 | 0 | 0.134388343 | TCGA-LB-A7SX-01 | 520 | 0 | 0.400343234 |
| TCGA-IB-A5SP-01 | 482 | 1 | 0.14474868 | TCGA-3E-AAAZ-01 | 2182 | 0 | 0.401091432 |
| TCGA-HV-A5A3-01 | 128 | 0 | 0.150834958 | TCGA-2J-AABH-01 | 1287 | 1 | 0.402346162 |
| TCGA-US-A779-01 | 511 | 0 | 0.16793768 | TCGA-HZ-A4BK-01 | 657 | 1 | 0.410862017 |
| TCGA-FB-AAQ1-01 | 123 | 0 | 0.188478801 | TCGA-FB-AAPQ-01 | 1130 | 0 | 0.413472597 |
| TCGA-2L-AAQM-01 | 1383 | 1 | 0.193179632 | TCGA-3A-A9IU-01 | 458 | 0 | 0.414260853 |
| TCGA-FB-AAQ2-01 | 153 | 0 | 0.195159117 | TCGA-2J-AABV-01 | 652 | 0 | 0.4163447 |
| TCGA-3A-A9IV-01 | 1103 | 1 | 0.206783276 | TCGA-IB-A5SS-01 | 460 | 0 | 0.426662917 |
| TCGA-IB-7644-01 | 741 | 0 | 0.209875994 | TCGA-IB-7654-01 | 476 | 0 | 0.426769756 |
| TCGA-HZ-A77O-01 | 171 | 0 | 0.212696272 | TCGA-IB-7889-01 | 481 | 0 | 0.427363972 |
| TCGA-3A-A9IJ-01 | 1854 | 1 | 0.223107882 | TCGA-IB-7651-01 | 603 | 0 | 0.429999994 |
| TCGA-HZ-7919-01 | 613 | 0 | 0.223906003 | TCGA-2J-AAB1-01 | 66 | 0 | 0.432141975 |
| TCGA-3A-A9IL-01 | 2741 | 1 | 0.233790564 | TCGA-2J-AABK-01 | 484 | 1 | 0.433929827 |
| TCGA-2J-AABU-01 | 277 | 0 | 0.237567524 | TCGA-3A-A9IZ-01 | 308 | 0 | 0.448619183 |
| TCGA-IB-7890-01 | 598 | 0 | 0.252081657 | TCGA-FB-A545-01 | 1117 | 0 | 0.456209447 |
| TCGA-US-A776-01 | 1216 | 1 | 0.266930382 | TCGA-FB-AAQ3-01 | 31 | 0 | 0.45975013 |
| TCGA-IB-A7LX-01 | 250 | 0 | 0.281056235 | TCGA-FB-A7DR-01 | 519 | 0 | 0.461667185 |
| TCGA-F2-6879-01 | 334 | 0 | 0.284542781 | TCGA-IB-7646-01 | 145 | 0 | 0.462158458 |
| TCGA-HZ-8005-01 | 120 | 0 | 0.28960488 | TCGA-3A-A9I5-01 | 1794 | 1 | 0.48849715 |
| TCGA-IB-A6UF-01 | 666 | 1 | 0.296710193 | TCGA-H8-A6C1-01 | 671 | 1 | 0.492195195 |
| TCGA-S4-A8RO-01 | 525 | 1 | 0.300090711 | TCGA-HV-A5A4-01 | 232 | 1 | 0.492206681 |
| TCGA-FB-AAPZ-01 | 716 | 1 | 0.318485904 | TCGA-IB-AAUO-01 | 239 | 0 | 0.49309352 |
| TCGA-3A-A9IH-01 | 1021 | 1 | 0.324129069 | TCGA-IB-7649-01 | 467 | 0 | 0.495831081 |
| TCGA-IB-A6UG-01 | 41 | 0 | 0.326831187 | TCGA-LB-A9Q5-01 | 468 | 0 | 0.497163769 |
| TCGA-FB-AAQ6-01 | 244 | 0 | 0.333348184 | TCGA-2J-AABT-01 | 319 | 1 | 0.498757007 |
| TCGA-FB-AAPU-01 | 381 | 0 | 0.335134726 | TCGA-YB-A89D-01 | 350 | 1 | 0.498988101 |
| TCGA-2J-AAB4-01 | 729 | 1 | 0.336427446 | TCGA-2J-AABI-01 | 969 | 1 | 0.518741059 |
| TCGA-2L-AAQA-01 | 143 | 0 | 0.34441192 | TCGA-XN-A8T3-01 | 951 | 1 | 0.5403719 |
| TCGA-2J-AAB9-01 | 627 | 0 | 0.345682009 | TCGA-2J-AABF-01 | 691 | 0 | 0.545123297 |
| TCGA-HZ-A8P1-01 | 7 | 1 | 0.345823051 | TCGA-L1-A7W4-01 | 442 | 0 | 0.545992891 |
| TCGA-F2-A7TX-01 | 95 | 0 | 0.346168027 | TCGA-US-A77E-01 | 430 | 0 | 0.548377413 |
| TCGA-HV-A7OP-01 | 978 | 1 | 0.350021739 | TCGA-US-A774-01 | 695 | 0 | 0.550620978 |
| TCGA-RB-AA9M-01 | 286 | 1 | 0.354146184 | TCGA-IB-7886-01 | 123 | 0 | 0.553715162 |
| TCGA-IB-AAUN-01 | 144 | 0 | 0.361535909 | TCGA-F2-A8YN-01 | 517 | 1 | 0.557734973 |
| TCGA-IB-7652-01 | 1116 | 1 | 0.367141236 | TCGA-M8-A5N4-01 | 584 | 1 | 0.56538622 |
| TCGA-2J-AAB6-01 | 293 | 0 | 0.368293145 | TCGA-FB-A78T-01 | 376 | 0 | 0.568048584 |
| TCGA-HZ-8002-01 | 390 | 0 | 0.578249255 | TCGA-IB-7885-01 | 1257 | 1 | 0.83168313 |
| TCGA-IB-A5SQ-01 | 219 | 0 | 0.580062649 | TCGA-3E-AAAY-01 | 2285 | 1 | 0.832355778 |
| TCGA-HV-A7OL-01 | 252 | 1 | 0.584724637 | TCGA-HV-AA8V-01 | 920 | 1 | 0.84685209 |
| TCGA-IB-8127-01 | 522 | 1 | 0.585212915 | TCGA-Q3-AA2A-01 | 95 | 1 | 0.85881221 |
| TCGA-F2-7273-01 | 952 | 0 | 0.588812073 | TCGA-HZ-7920-01 | 236 | 0 | 0.872623987 |
| TCGA-HZ-8638-01 | 242 | 0 | 0.592520264 | TCGA-IB-7645-01 | 1502 | 0 | 0.883471801 |
| TCGA-IB-7887-01 | 110 | 0 | 0.595443671 | TCGA-IB-AAUM-01 | 8 | 1 | 0.891647685 |
| TCGA-IB-AAUU-01 | 245 | 1 | 0.599855954 | TCGA-3A-A9J0-01 | 743 | 1 | 0.912580054 |
| TCGA-S4-A8RP-01 | 702 | 0 | 0.603991812 | TCGA-RL-AAAS-01 | 9 | 1 | 0.912658562 |
| TCGA-HZ-A49G-01 | 660 | 1 | 0.608253987 | TCGA-HZ-8519-01 | 454 | 1 | 0.926839384 |
| TCGA-HZ-8317-01 | 394 | 0 | 0.608918006 | TCGA-HZ-8315-01 | 327 | 0 | 0.939182552 |
| TCGA-HZ-7922-01 | 4 | 1 | 0.609523066 | TCGA-2J-AABA-01 | 607 | 0 | 0.947549029 |
| TCGA-XD-AAUL-01 | 498 | 1 | 0.616917019 | TCGA-IB-AAUW-01 | 409 | 0 | 0.950050321 |
| TCGA-2L-AAQL-01 | 292 | 0 | 0.621633349 | TCGA-RB-A7B8-01 | 502 | 0 | 1.004206134 |
| TCGA-IB-A7M4-01 | 483 | 1 | 0.622452779 | TCGA-H6-A45N-01 | 654 | 0 | 1.013089616 |
| TCGA-FB-AAPY-01 | 1059 | 0 | 0.625959575 | TCGA-HZ-A4BH-01 | 194 | 1 | 1.021742026 |
| TCGA-IB-7893-01 | 117 | 0 | 0.633657353 | TCGA-2J-AABR-01 | 438 | 1 | 1.025515499 |
| TCGA-2J-AABO-01 | 440 | 1 | 0.63664412 | TCGA-OE-A75W-01 | 377 | 0 | 1.042421093 |
| TCGA-FB-AAQ0-01 | 473 | 0 | 0.636912538 | TCGA-IB-AAUT-01 | 287 | 1 | 1.057276499 |
| TCGA-2L-AAQE-01 | 684 | 0 | 0.646186736 | TCGA-YH-A8SY-01 | 388 | 1 | 1.095662651 |
| TCGA-HZ-A77P-01 | 330 | 1 | 0.65017534 | TCGA-2J-AAB8-01 | 80 | 1 | 1.110041577 |
| TCGA-HZ-8636-01 | 550 | 0 | 0.652350119 | TCGA-US-A77J-01 | 568 | 0 | 1.151032199 |
| TCGA-IB-7888-01 | 1332 | 0 | 0.653391478 | TCGA-2J-AABP-01 | 463 | 1 | 1.158623586 |
| TCGA-3A-A9I7-01 | 1323 | 1 | 0.654039332 | TCGA-3A-A9IX-01 | 1037 | 1 | 1.269771669 |
| TCGA-2J-AABE-01 | 676 | 1 | 0.674734358 | TCGA-HZ-7925-01 | 975 | 0 | 1.305387218 |
| TCGA-HV-A5A5-01 | 289 | 1 | 0.684044354 | TCGA-IB-A5ST-01 | 635 | 1 | 1.336395544 |
| TCGA-3A-A9IC-01 | 738 | 0 | 0.688327946 | TCGA-IB-A5SO-01 | 694 | 0 | 1.340769381 |
| TCGA-F2-A44G-01 | 386 | 0 | 0.690865949 | TCGA-IB-8126-01 | 462 | 1 | 1.359912442 |
| TCGA-FB-A4P5-01 | 183 | 0 | 0.692407449 | TCGA-XD-AAUH-01 | 395 | 1 | 1.38954847 |
| TCGA-HZ-7924-01 | 840 | 1 | 0.705568979 | TCGA-HZ-A49H-01 | 491 | 1 | 1.410819646 |
| TCGA-F2-7276-01 | 216 | 0 | 0.716605399 | TCGA-XN-A8T5-01 | 720 | 1 | 1.464534126 |
| TCGA-IB-7891-01 | 1561 | 0 | 0.72059436 | TCGA-HZ-8637-01 | 517 | 0 | 1.533823376 |
| TCGA-IB-AAUQ-01 | 183 | 0 | 0.721775054 | TCGA-HZ-7923-01 | 314 | 1 | 1.594219479 |
| TCGA-HZ-8003-01 | 617 | 0 | 0.734557384 | TCGA-IB-7897-01 | 486 | 0 | 1.627243663 |
| TCGA-3A-A9IB-01 | 224 | 0 | 0.74878805 | TCGA-XD-AAUG-01 | 420 | 1 | 1.774382311 |
| TCGA-F2-A44H-01 | 586 | 1 | 0.750963892 | TCGA-HZ-8001-01 | 706 | 1 | 1.848753858 |
| TCGA-XD-AAUI-01 | 568 | 0 | 0.76206829 | TCGA-IB-AAUP-01 | 431 | 1 | 2.00809027 |
| TCGA-2L-AAQJ-01 | 394 | 0 | 0.773891385 | TCGA-HZ-A77Q-01 | 33 | 1 | 2.183384707 |
| TCGA-FB-A5VM-01 | 947 | 0 | 0.77713927 | TCGA-IB-AAUR-01 | 338 | 1 | 2.319819756 |
| TCGA-3A-A9I9-01 | 634 | 0 | 0.787327579 | TCGA-FB-AAPS-01 | 228 | 1 | 2.585755359 |
| TCGA-HZ-7918-01 | 969 | 1 | 0.814925128 | TCGA-Q3-A5QY-01 | 416 | 1 | 2.634613826 |
| TCGA-HZ-A49I-01 | 308 | 0 | 0.817079522 | TCGA-Z5-AAPL-01 | 467 | 1 | 2.644544014 |
| TCGA-PZ-A5RE-01 | 717 | 0 | 0.819552237 | TCGA-IB-AAUS-01 | 225 | 1 | 2.989856447 |
| TCGA-FB-A4P6-01 | 767 | 1 | 0.823526758 | TCGA-IB-AAUV-01 | 404 | 1 | 3.092822273 |

**2. Supplementary Figures:**

**2.1. Supplementary Fig. S1.** Flow chart of the research process in this study.


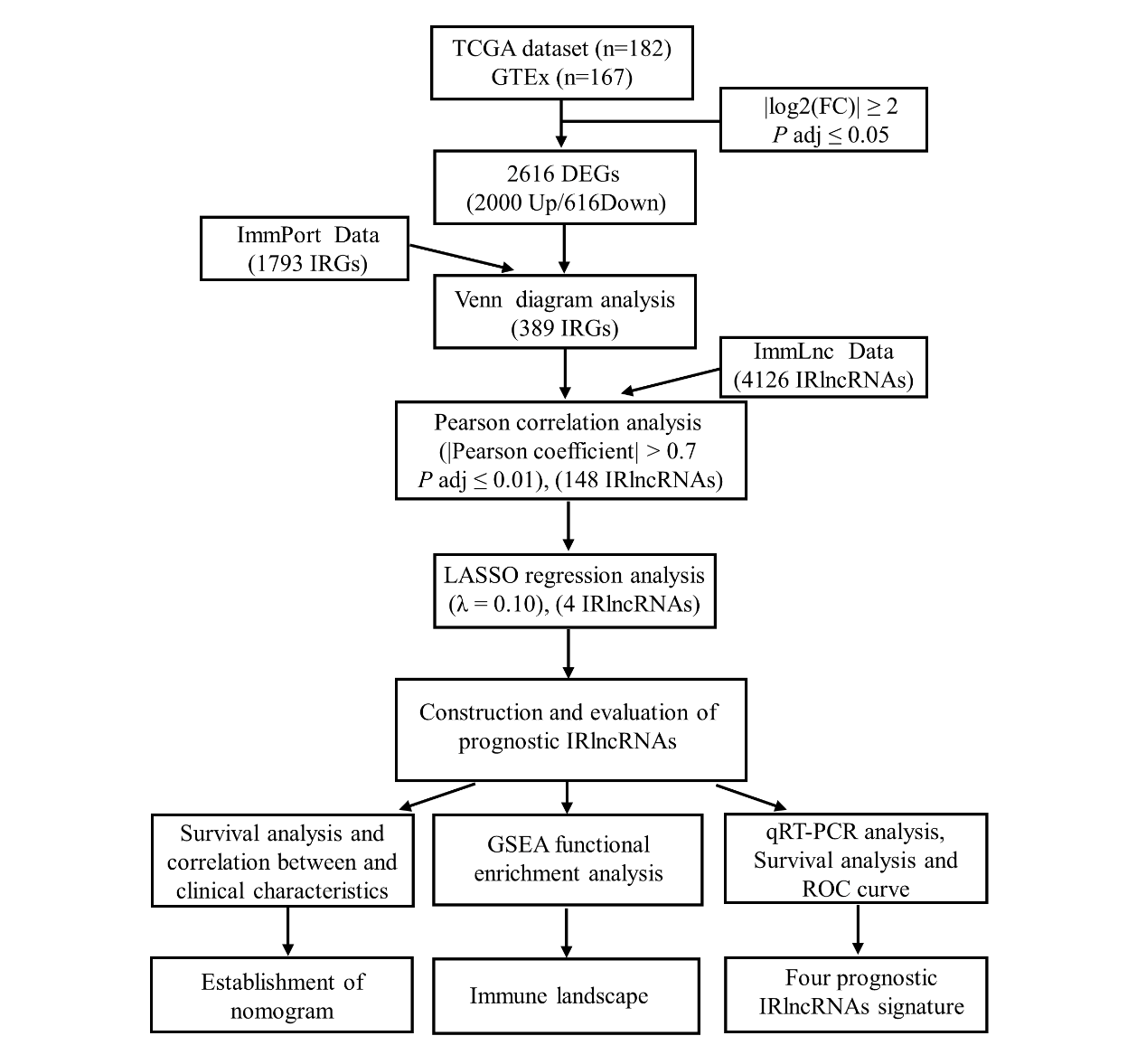


**2.2. Supplementary Fig. S2.** Plot diagrams showing the distribution of patients in different risk groups. **(A)** t-SNE plot**. (B)** UMAP plot.


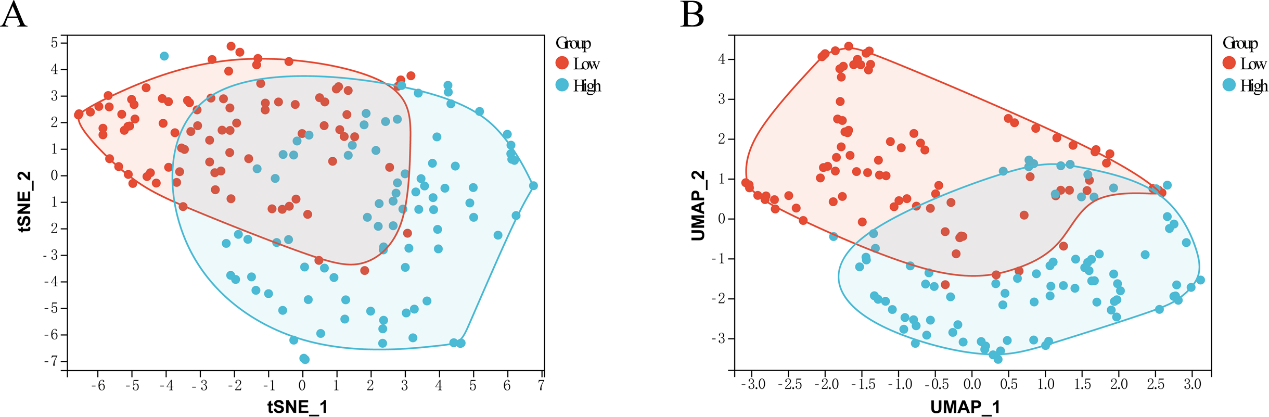


**2.3. Supplementary Fig. S3.** Different levels of risk scores in PaCa patients were stratified by gender, age, and stage **(A-C).**


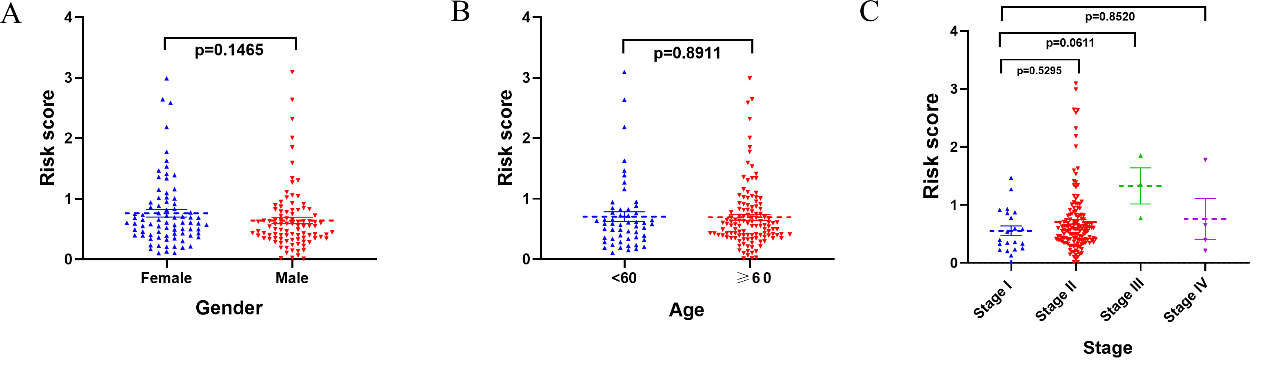


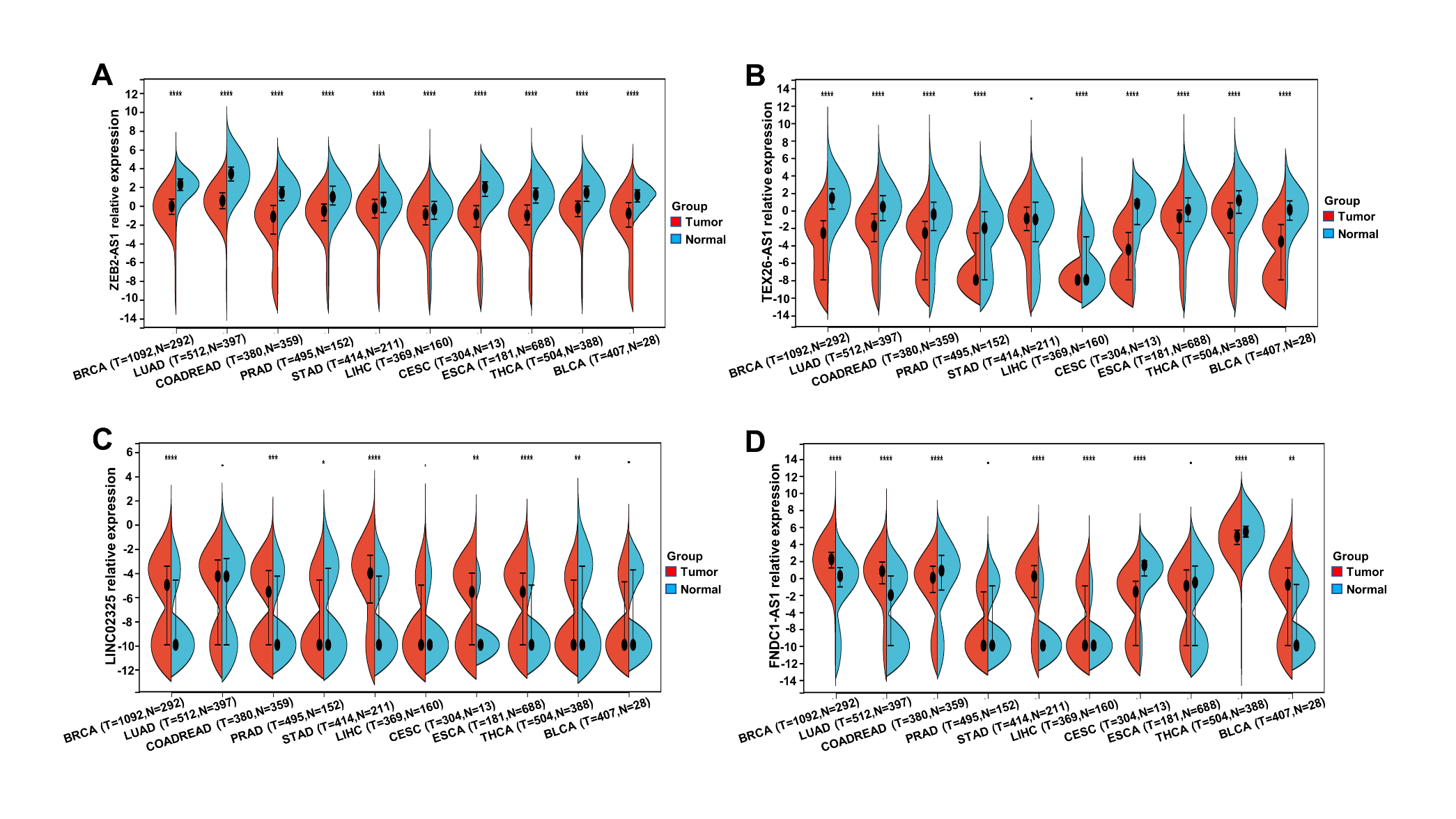
**2.4. Supplementary Fig. S4.** The four IRlncRNAs expression levels in the ten most frequent cancer in 2020. (**A**) ZEB2-AS1; (**B**) TEX26-AS1; (**C**) LINC02325; (**D**) FNDC1-AS1.

**2.5. Supplementary Fig. S5.** Correlation between four IRlncRNAs expression levels and IRGs expression levels in PaCa. (**A**) LINC02325 and CD3E; (**B**) FNDC1-AS1 and NOX4; (**C**) TEX26-AS1 and THBS1; (**D**) ZEB2-AS1 and GMFG.


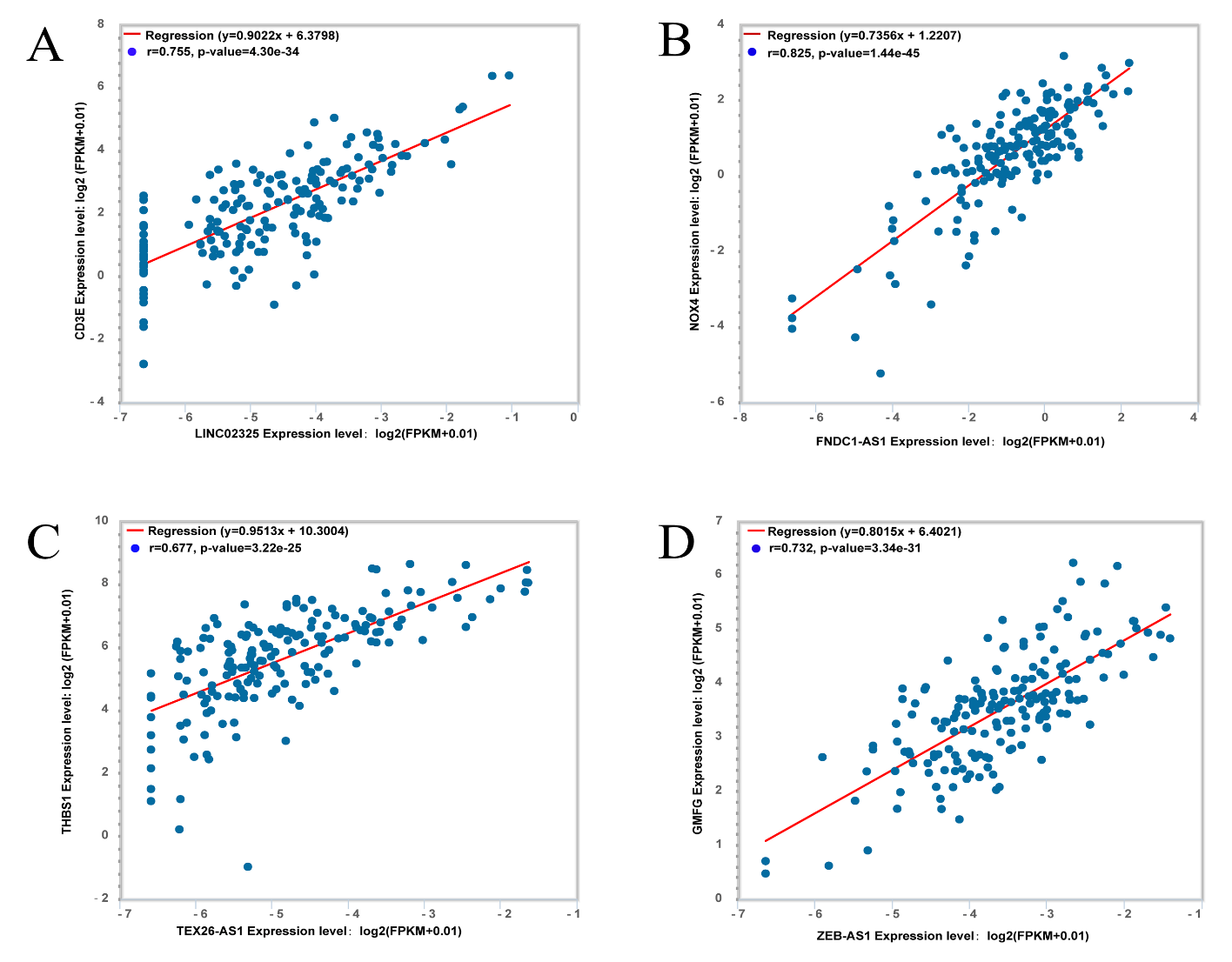


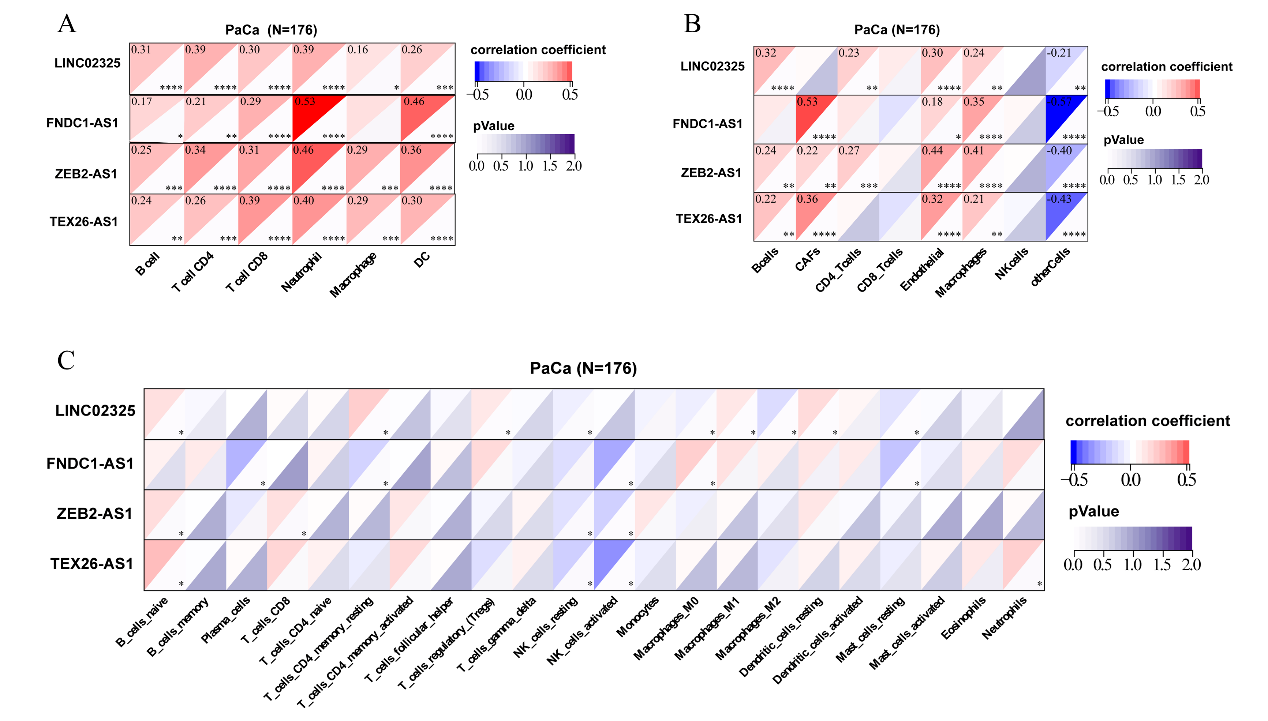
**2.6. Supplementary Fig. S6.** Estimation of tumor-infiltrating immune cells based on TIMER algorithms (**A**), EPIC algorithms (**B**) and CIBERSORT algorithms (**C**). The X-axis represents the type of immune cells, and the Y-axis represents four lncRNAs. The numbers in the top left represent the correlation coefficients. Red means positive correlation, while blue means negative correlation. The darker color stands for a stronger correlation.

**7. Supplementary Fig. S7.** Estimation of tumor-infiltrating immune cells based on MCPCOUNTER algorithms (**A**) and XCELL algorithms (**B**).


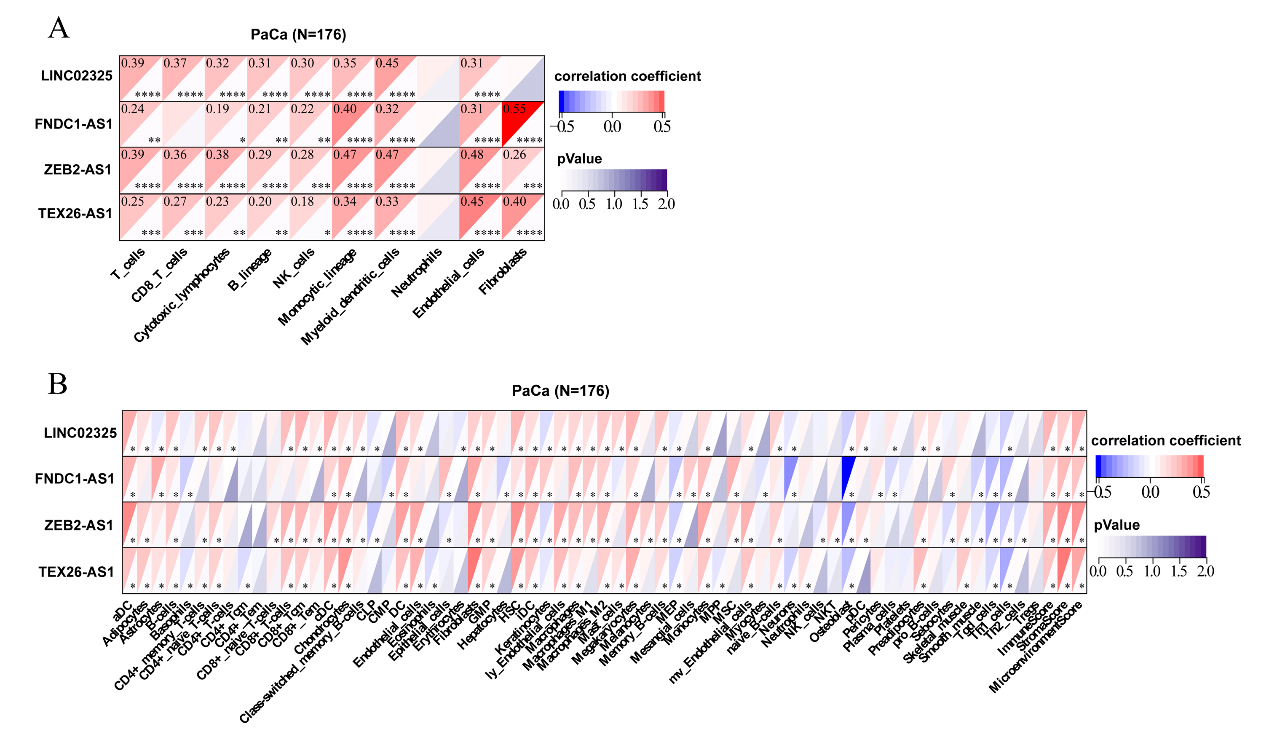


**2.8. Supplementary Fig. S8.** Estimation of tumor-infiltrating immune cells based on QUANTISEQ algorithms (**A**) and IPS algorithms (**B**). Correlation analysis of thirty-four immune checkpoints and four lncRNAs expression (**C**).


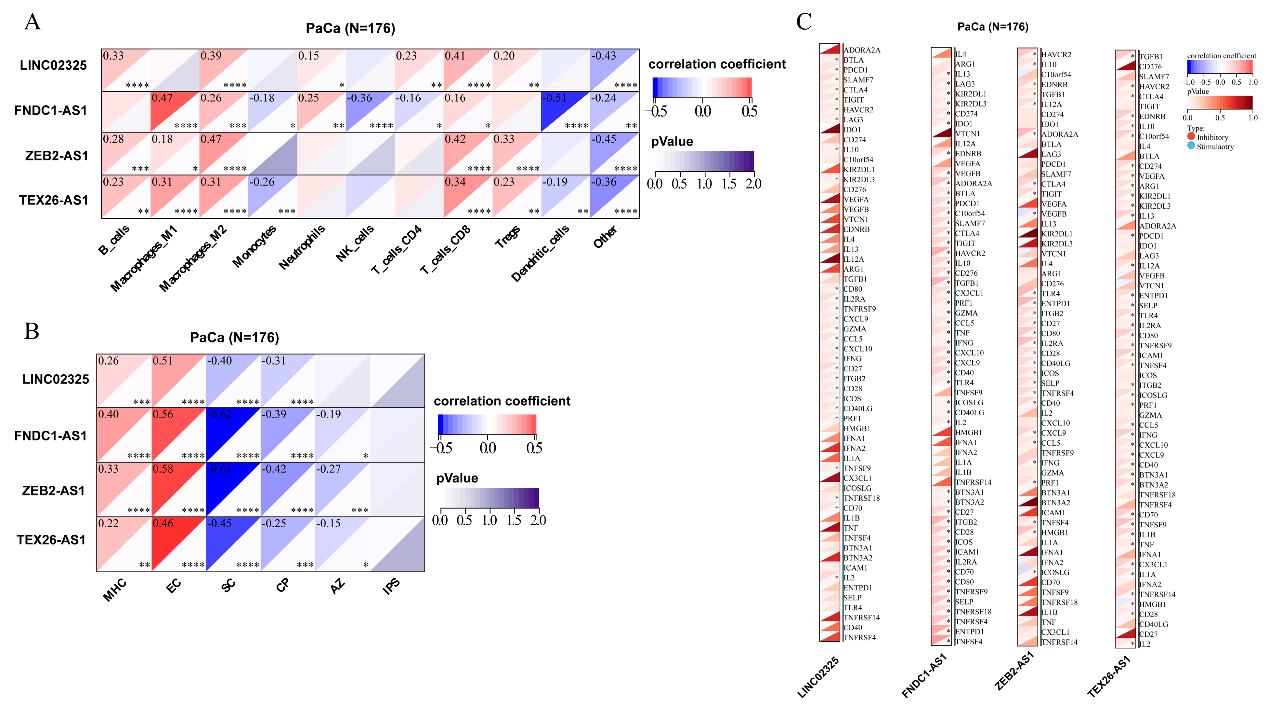


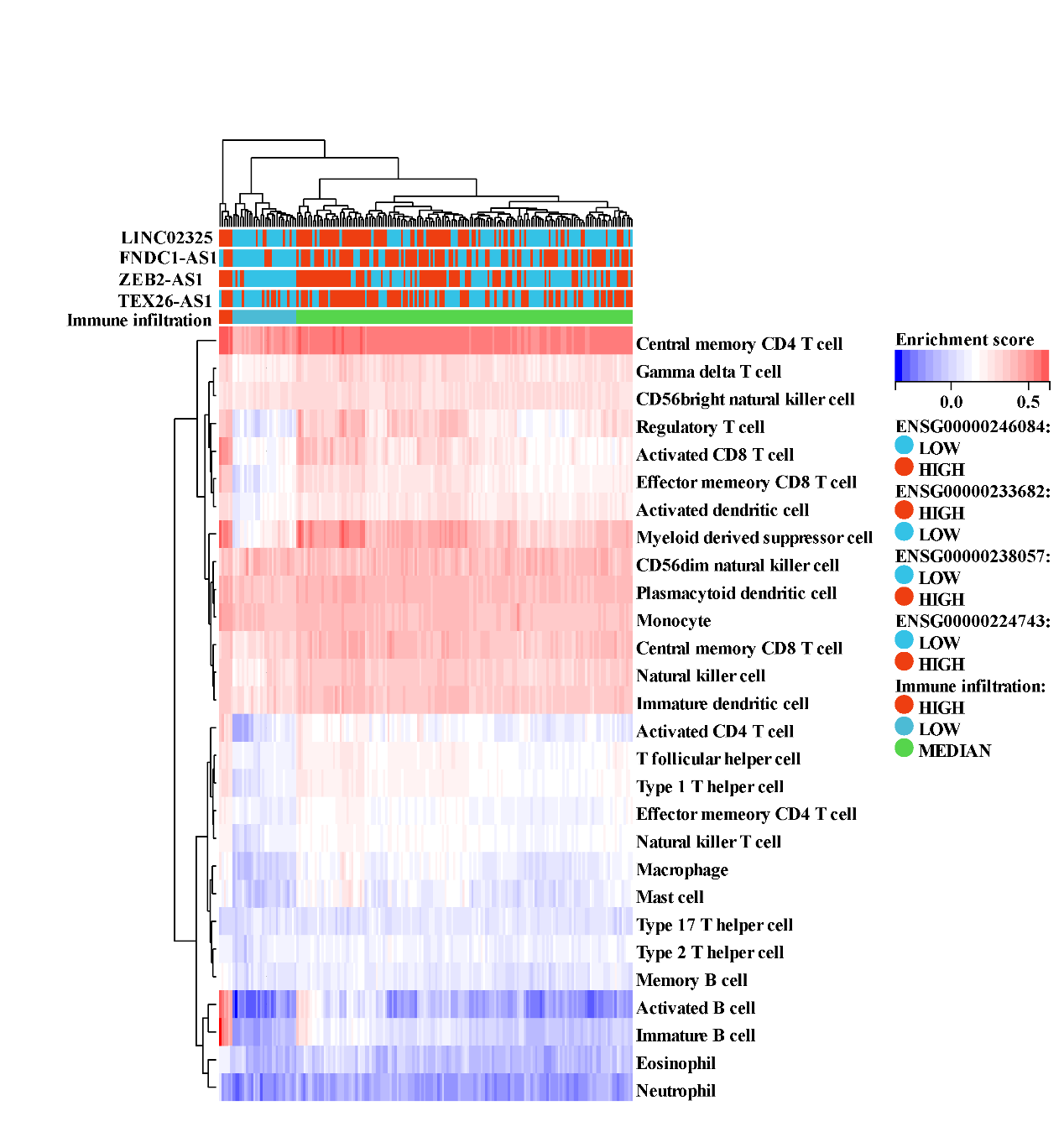
**2.9. Supplementary Fig. S9.** Construction of immune cell infiltration groups in PaCa. The enrichment levels of 28 immune-related cells and types in high immune infiltration, median immune cell infiltration and low immune cell infiltration, and the expression levels of four IRlncRNA was combined with clustering information.


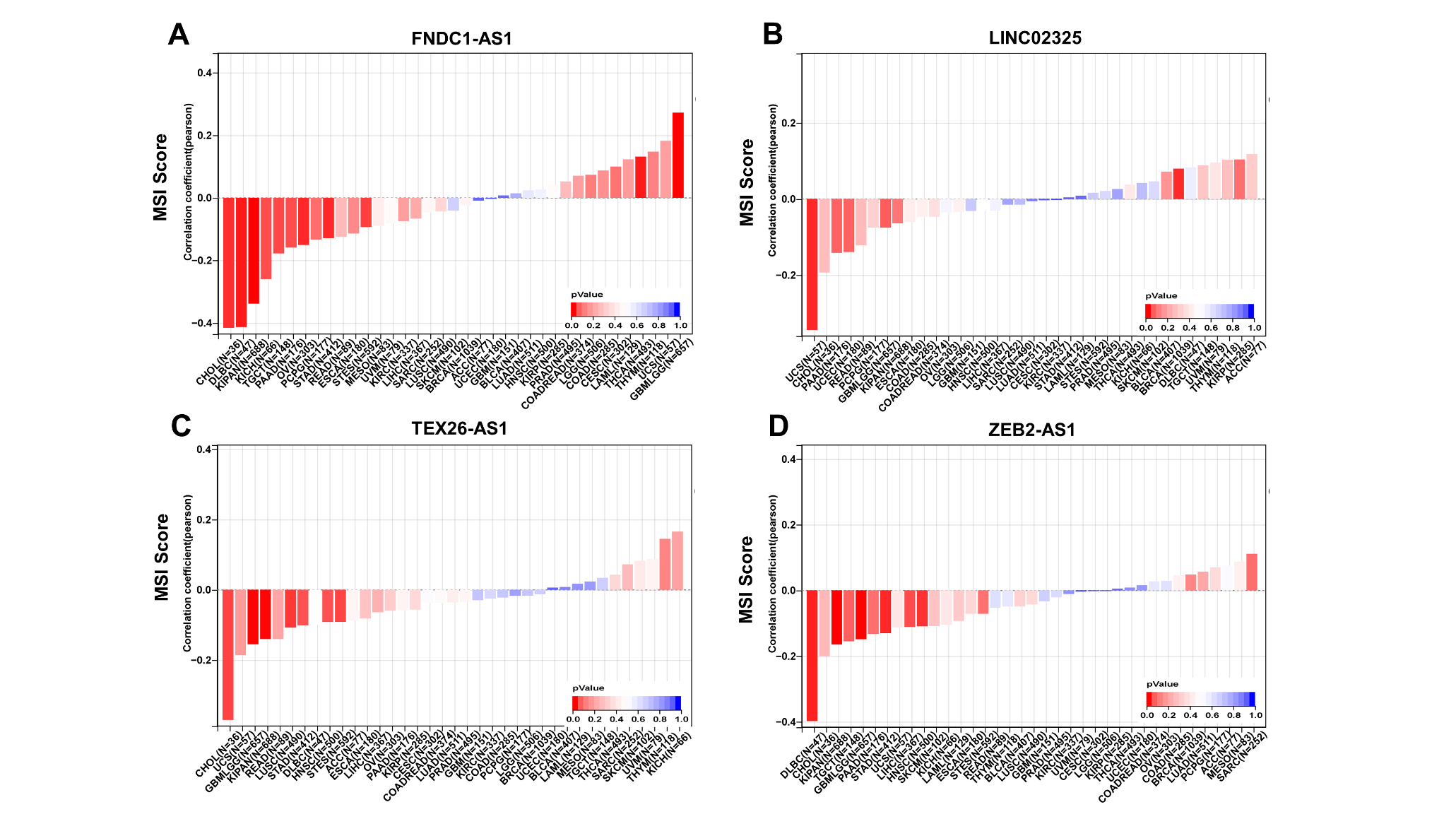
**2.10. Supplementary Fig. S10** Correlation between four IRlncRNAs expressions levels and MSI scores. (**A**) FNDC1-AS1; (**B**) LINC02325; (**C**) TEX26-AS1; (**D**) ZEB2-AS1.


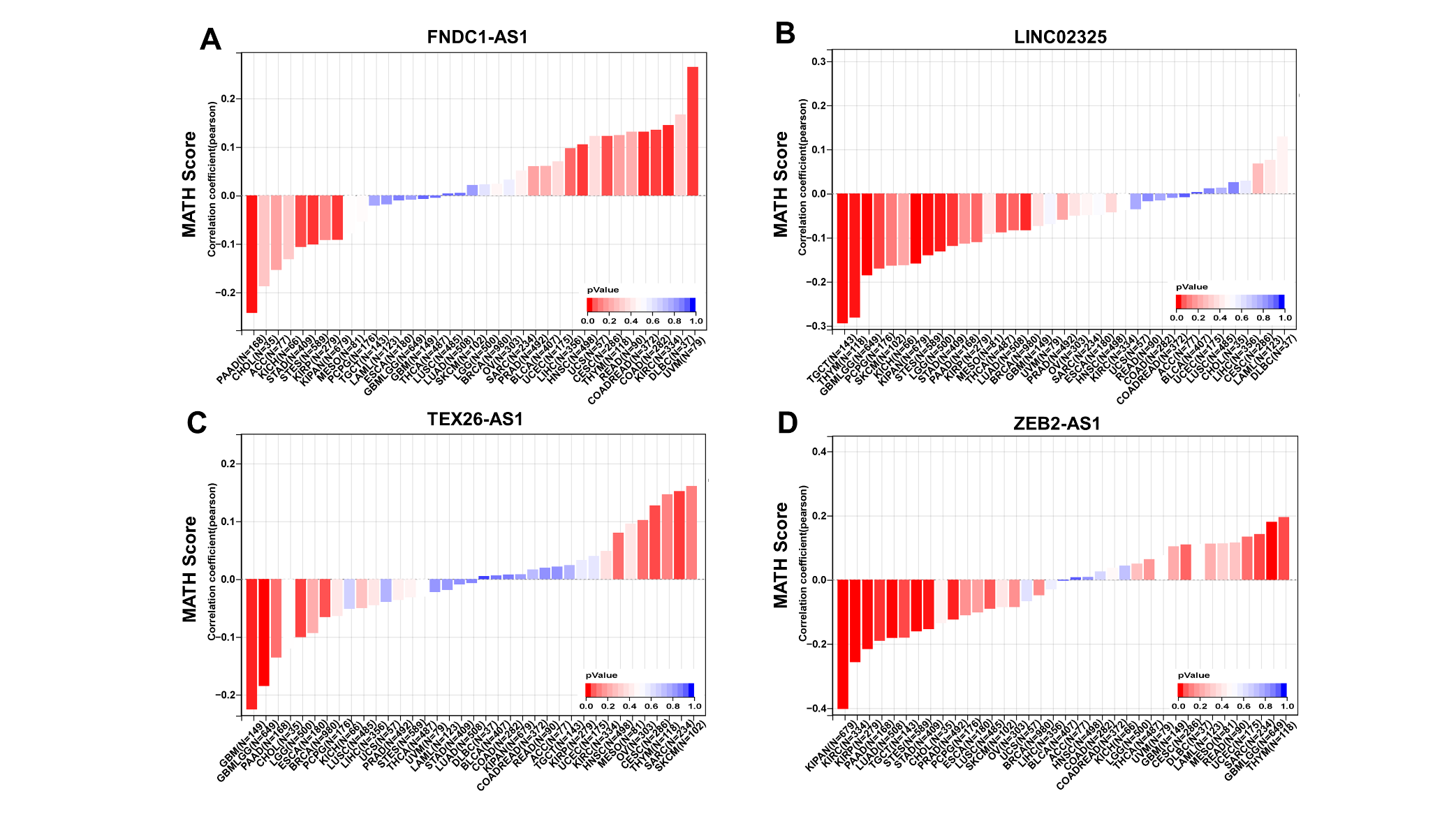
**2.11. Supplementary Fig. S11** Correlation between four IRlncRNAs expressions levels and MATH scores. (**A**) FNDC1-AS1; (**B**) LINC02325; (**C**) TEX26-AS1; (**D**) ZEB2-AS1.
